# Characterization of Myelomonocytoid Progenitor Cells with Mesenchymal Differentiation Potential Obtained by Outgrowth from Pancreas Explants

**DOI:** 10.1155/2012/429868

**Published:** 2012-08-21

**Authors:** Marc-Estienne Roehrich, Giuseppe Vassalli

**Affiliations:** ^1^Department of Cardiology, Centre Hospitalier Universitaire Vaudois (CHUV), Avenue du Bugnon, 1011 Lausanne, Switzerland; ^2^Molecular Cardiology Laboratory, Fondazione Cardiocentro Ticino, via Tesserete 48, 6900 Lugano, Switzerland

## Abstract

Progenitor cells can be obtained by outgrowth from tissue explants during primary ex vivo tissue culture. We have isolated and characterized cells outgrown from neonatal mouse pancreatic explants. A relatively uniform population of cells showing a distinctive morphology emerged over time in culture. This population expressed monocyte/macrophage and hematopoietic markers (CD11b^+^ and CD45^+^), and some stromal-related markers (CD44^+^ and CD29^+^), but not mesenchymal stem cell (MSC)-defining markers (CD90^−^ and CD105^−^) nor endothelial (CD31^−^) or stem cell-associated markers (CD133^−^ and stem cell antigen-1; Sca-1^−^). Cells could be maintained in culture as a plastic-adherent monolayer in culture medium (MesenCult MSC) for more than 1 year. Cells spontaneously formed sphere clusters “pancreatospheres” which, however, were nonclonal. When cultured in appropriate media, cells differentiated into multiple mesenchymal lineages (fat, cartilage, and bone). Positive dithizone staining suggested that a subset of cells differentiated into insulin-producing cells. However, further studies are needed to characterize the endocrine potential of these cells. These findings indicate that a myelomonocytoid population from pancreatic explant outgrowths has mesenchymal differentiation potential. These results are in line with recent data onmonocyte-derivedmesenchymal progenitors (MOMPs).

## 1. Introduction 

The pancreas is a complex organ consisting of three principal cell types: endocrine islets, exocrine acini, and ducts. Evidence of differentiation of new *β*-cells from pancreatic nonislet cells suggests the existence of pancreatic nonendocrine stem/progenitor cells [1, 2]. New *β*-cells may also result from replication of preexisting *β*-cells [3], or from progenitor cells originating from the ductal epithelium [4–6] or the exocrine tissue of the pancreas [7–9]. Pancreatic progenitor cells express key transcription factors involved in the embryological development of endocrine cells such as pancreatic and duodenal homeobox factor 1 (Pdx1), neurogenin 3 (Ngn3) and paired box 4 (Pax4), or embryonic markers such as Oct-4 and Nanog, or nestin [10]. Pancreatic progenitor cells have been prospectively isolated by fluorescence-activated cell sorting (FACS) using specific antibodies that recognize cell-surface epitopes expressed by stem/progenitor cells in other tissues, such as CD133, CD117 (c-kit/stem cell factor receptor), ATP-binding cassette (ABC) G2, and mesenchymal stem cell (MSC) markers [11–15].

An alternative method for the isolation of tissue-resident progenitor cells is the explant outgrowth approach. This method does not rely on positive cell selection. Within tissue explants, progenitor cells are located in close proximity to stem cell niches, which regulate stem and progenitor cell function [16]. These cells may become activated during *ex vivo *primary tissue culture, migrate across chemotactic gradients towards the surface of the tissue explant, are shed by it, and form a monolayer. Primary tissue cultures of the adult or embryonic pancreas have been described extensively [17]. In contrast, data on pancreas explant cell outgrowths are limited. Using the explant outgrowth technique, Schneider et al. [18] isolated stellate cells from pancreata of rats with cerulein pancreatitis. Bläuer et al. [19] designed a new explant outgrowth system that allowed for the isolation of pancreatic acinar cells at the gas-liquid interphase. Carlotti et al. [20] reported that the cell outgrowth from isolated human islets was comprised of adherent fibroblastoid cells that expressed MSC and pericyte markers, as well as nestin and vimentin, but not genes for endocrine hormones. When cultured under appropriate conditions, these cells differentiated into adipocytes and osteoblasts lineages and expressed insulin, glucagons, and somatostatin genes. Several other studies attempting to generate *β*-cells from precursor cells from endocrine or exocrine pancreatic explants documented the presence of plastic-adherent mesenchymal cells in cell cultures [21–25]. While early studies suggested that epithelial-to-mesenchymal transition by *β*-cells might be responsible for the occurrence of these mesenchymal cells [21], this assumption was recently refuted based on lineage tracing experiments [26–29].

MSCs are multipotent precursor cells for stromal cells, which are capable of differentiating into multiple ectodermal, mesodermal, and endodermal tissues [30]. As such, they have been considered a source of cells for therapeutic approaches for various conditions, including type-1 diabetes. Experimental evidence suggests bone marrow (BM), or adipose tissue-derived MSCs are capable of differentiating into insulin-producing cells *in vitro* and contribute to the restoration of normoglycemia in animal models of diabetes *in vivo* [31, 32]. Human mesenchymal stromal cells that differentiate and mature to hormone-expressing cells *in vivo* have been referred to as islet-derived precursor cells (IPCs) [33]. Recent evidence suggests MSCs may act as trophic mediators to attenuate *β*-cell death and activate endogenous regenerative mechanisms [34–41].

The present study aimed to characterize the mouse pancreas explant cell outgrowth during *ex vivo* tissue culture. Unlike Carlotti et al. [20] who studied islet outgrowths, we used whole pancreas explants. We reproducibly obtained a population of cells that exhibited a relatively uniform morphology and a stable cell-surface marker profile. The latter was characterized by expression of monocyte/macrophage and hematopoietic markers (CD11b and CD45), pericyte/perivascular markers (neuron-glial antigen 2 [NG2] proteoglycan and, to a lesser extent, CD146) [42], and certain MSC and/or endothelial progenitor cell (EPC) markers (CD29 and CD44), but not MSC-defining (CD90 and CD105) and endothelial (CD31) markers. The isolated myelomonocytoid population was propagated for up to 5 passages and was maintained in culture as a monolayer for more than 1 year with no major morphologic or immunophenotypic changes. Plastic-adherent cells spontaneously formed spherical clusters that detached from plastic, which is considered a feature of stemness [43]. They were capable of differentiating along multiple mesenchymal lineages (fat, cartilage, and bone) although this was not demonstrated with single-cell cloning. These findings indicate that pancreas explant cell outgrowths can give rise to a myelomonocytoid population endowed with mesenchymal differentiation potential. These findings are inline with recent data on monocyte-derived mesenchymal progenitors (MOMPs) [44].

## 2. Materials and Methods

### 2.1. Cell Isolation and Culture

Pancreatic explants were obtained from neonatal (1-2 days of age) male C57Bl/6 mice (from Charles River Laboratories, France) or C57BL/6-Tg(CAG-EGFP)1Osb/J transgenic mice expressing enhanced green fluorescent protein (EGFP) from an immediate-early CMV promoter (gift of T. Pedrazzini, CHUV, Lausanne). Tissue explants were rinsed abundantly with heparinized saline and then cut into small pieces that were placed in Corning Costar 6-well culture plates (Sigma) with no extracellular matrix (EMC) protein coating. Explants were cultured in MesenCult (MesenCult MSC Basal Medium [Mouse] supplemented with serum-containing MesenCult MSC Stimulatory Supplements [Mouse], both from Stem Cell Technologies). After 2 weeks, tissue explants were removed from the culture plates, while the cell outgrowth was left in place. When adherent cells formed a nearly confluent monolayer, they were detached from plastic with PBS-EDTA, collected, and seeded onto new plates. In separate experiments (*n* = 2), cells were cultured in Dulbecco-modified Eagle medium supplemented with 10% fetal calf serum (DMEM-10% FCS), with or without granulocyte-macrophage colony-stimulating factor (GM-CSF). In a separate experiment, cells were cultured using a MethoCult (Stem Cell Technologies)-based 3D system.

### 2.2. Flow Cytometric Analyses

For flow cytometric analyses (*n* = 6), cells were gently detached from plastic with PBS-EDTA, filtered through a 70-*μ*m filter, centrifuged, and resuspended in reagent A Leucoperm B4FO9B (AbD Serotec) for 15 min at RT. Then, PBS was added, and cells were centrifuged, resuspended in reagent B, incubated with primary antibody (see Supplementary Table 1 available online at doi:10.1155/2012/429868) for 30 min at RT, and washed with PBS. When needed, cells were resuspended in reagent B and incubated with mouse anti-rat Alexa 488 (1 : 25 dilution) for 30 min at RT. Flow cytometric analyses were performed using a FACSCalibur system (BD Bioscience) and the CellQuest software. Gates used to resolve antigen-expressing cells were set using appropriate isotype-specific control antibodies.

### 2.3. Immunocytochemistry

Immunocytochemistry was performed as previously described [45]. Briefly, cells were seeded on Lab-Tek Chamber-Slides (Nunc) and fixed with 1% paraformaldehyde (PFA). For immunostaining of NG2 proteoglycan, a polyclonal rabbit anti-NG2 antibody (Chemicon/Millipore) followed by a goat-anti-rabbit secondary Ab labeled with Alexa 488 (Invitrogen; 1 : 400 dilution) was used. Nuclei were stained with DAPI.

### 2.4. Sphere Formation and Clonogenicity

Free-floating spherical clusters formed spontaneously from monolayers of plastic-adherent cells plated on Corning Costar 6-well plates (*n* = 2 experiments). To assess whether spherical cell clusters were clonally derived, mixtures of pancreatic cell outgrowths from C57BL/6 wildtype (WT) and from C57BL/6-Tg(CAG-EGFP) 1Osb/J transgenic mice were cultured at varying cell ratios. Spheres were analyzed for green fluorescent areas under the fluorescence microscope after 3 weeks (*n* = 1 experiment).

### 2.5. Differentiation Assays and Cell Staining

To induce adipogenic, osteogenic, and chondrogenic differentiation, pancreatic outgrowth-derived cells were cultured for 3 months in MesenCult and then changed to NH AdipoDiff, OsteoDiff, and ChondroDiff Media (all from Miltenyi), respectively, for 17 days. Adipogenic differentiation was detected by incubating cells with 1% PFA for 10 min, followed by Oil red-O solution for 15 min, and three PBS washes. Osteogenic differentiation was detected by incubating cells with 1% PFA for 10 min, followed by 2% Alizarin red for 5 min, and three PBS washes. Chondrogenic differentiation was detected by staining cells with Alcian-blue. For detection of pancreatic *β*-cells, cells cultured in MesenCult supplemented with 1.27 *μ*M dexamethasone for 18 days were incubated with 1% PFA for 10 min, and stained with the zinc-chelating agent, dithizone (Merck) [46–49], for 15 min according to the manufacturer's instructions.

## 3. Results

### 3.1. Cell Morphology and Culture

**Figure 1:**
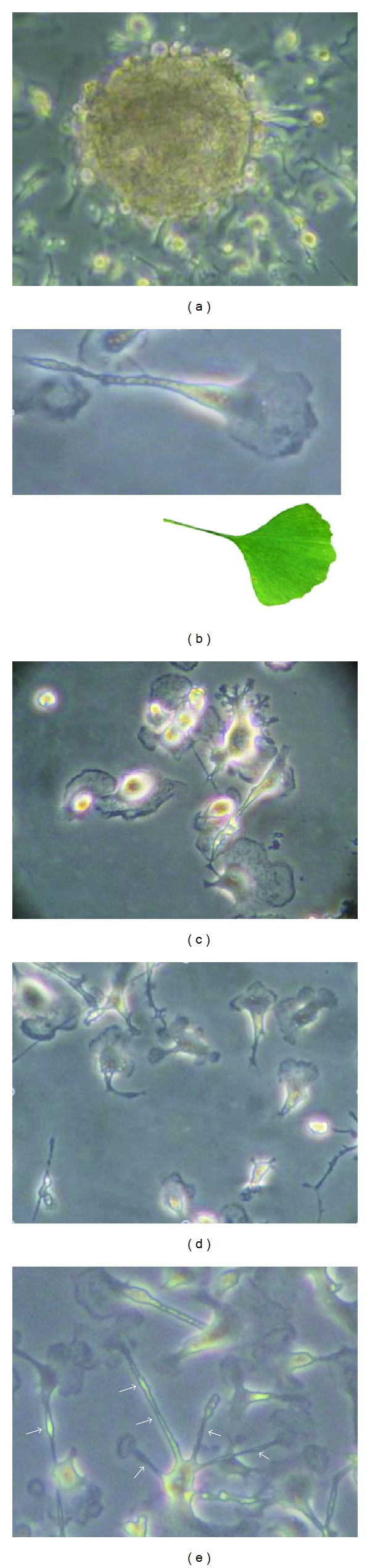
(a) Phase photomicrograph of a pancreatic explant with outgrowing cells in the primary *ex vivo* tissue culture. (b) High magnification view of an expanded pancreas-derived cell showing a characteristic *Gingko biloba* leaf-like shape (insert). (c and d) Expanded pancreas-derived cells showing refringent nuclei and thin cytoplasmic processes. (e) Expanded pancreas-derived cells showing a “stellate” pattern of cytoplasmic processes (arrows) with knobs on their extremities.

Using the explant outgrowth approach, cells shed by cultured pancreatic explants from neonatal mice were observed on day 3-4, initially as a heterogeneous population of plastic-adherent cells showing both spindle-shaped and round morphologies (Figure 1(a)). After 4–6 weeks, cells acquired a relatively uniform morphology characterized by one or multiple thin cytoplasmic processes carrying a knob on their extremities (this distinctive cell morphology resembled a *gingko biloba* leaf; Figures 1(b) and 1(d)). Cells with multiple thin processes exhibited a stellate shape, whereby processes from neighboring cells appeared to establish inter-cellular contacts (Figure 1(e)). Cells grew slowly and were propagated for up to 5 passages. They could be maintained in culture for more than 1 year.

### 3.2. Cell-Surface Marker Profile

**Figure 2:**
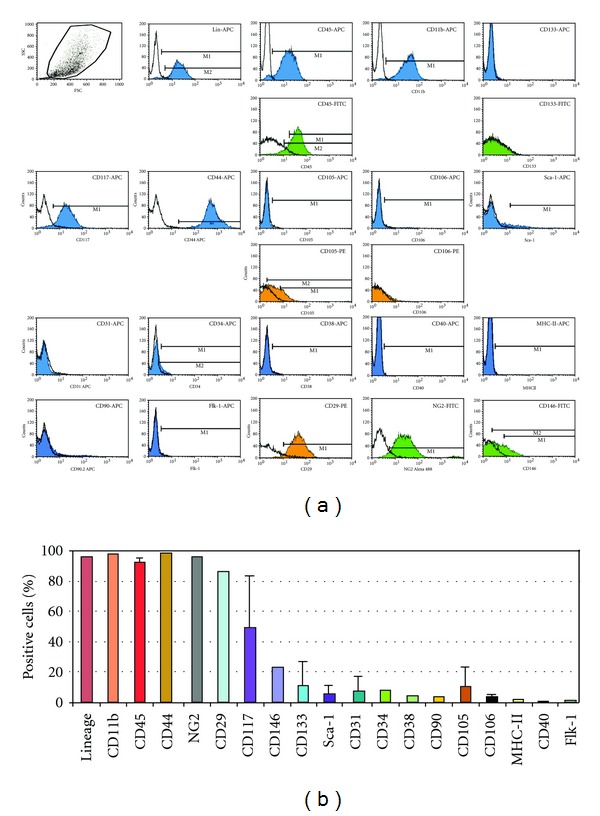
Flow cytometric analyses of the cell-surface marker profile of pancreas outgrowth-derived cells. (a) Representative analysis of cell-surface marker expression of cells cultured 2 months in MesenCult. Blue, green, and orange colors indicate APC, FITC, and PE fluorochromes, respectively. Selected markers (CD45, CD105, CD106, and CD133) were determined with two different fluorochromes. (b) Mean percentages (±SD) of cells expressing the indicated cell-surface markers (*n* = 6 analyses; 3–5 samples for each marker, excepted for a subset of markers [NG2, CD29, CD146, CD34, CD38, MHC-II, CD40, and Flk-1] for which a single measure is available).

**Figure 3:**
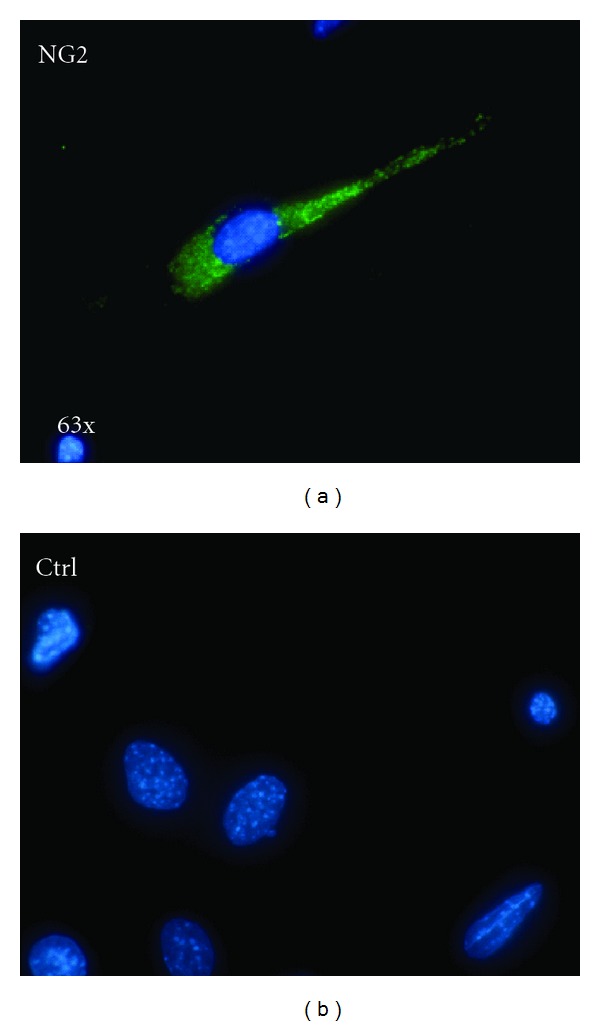
NG2 expression by pancreas outgrowth-derived cells. (a) NG2 immunostaining using an Alexa 488-labeled secondary antibody (green); nuclear staining with DAPI (blue). (b) Control (secondary antibody only).

**Figure 4:**
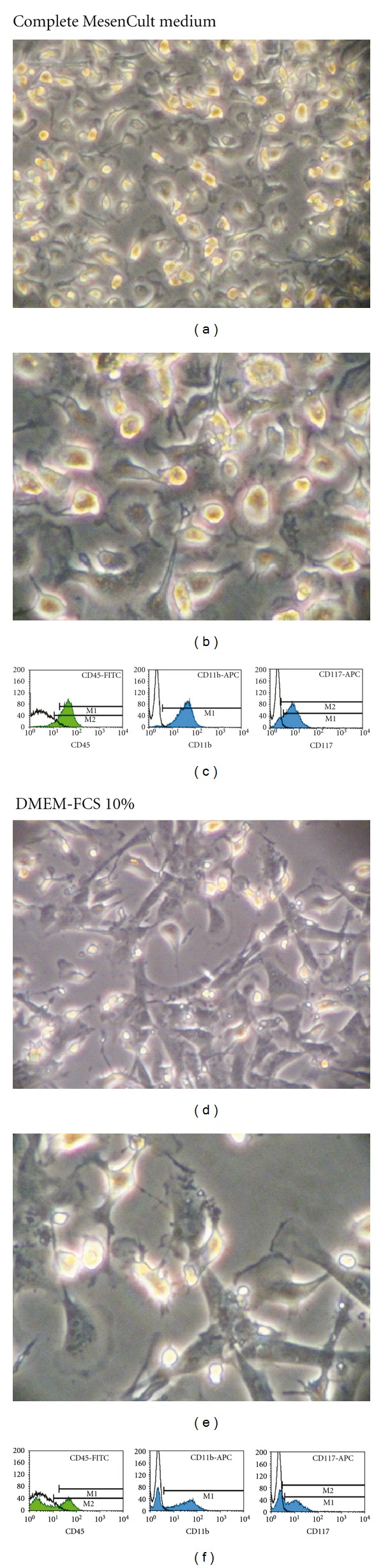
Effects of the culture medium on cell morphology and antigen expression. (a–c) Cells cultured in MesenCult for 1 month display highly refringent nuclei, *gingko biloba* leaf-like shapes, and CD45, CD11b, and CD117 expression. (d–f) Cells initially derived in MesenCult and then changed to DMEM-FCS 10% show less refringent nuclei, rhomboid shapes, and decreased subsets of CD45^+^, CD11b^+^, or CD117^+^ cells.

Cells cultured for 2 months in MesenCult were analyzed by flow cytometry. They expressed lineage (Lin) differentiation antigens, the common leukocyte antigen CD45, the monocytic marker CD11b, the hematopoietic marker c-kit (CD117), the pericyte/mesoangioblast markers NG2 proteoglycan, and CD146 [24], CD44 (a receptor for hyaluronic acid considered a MSC and EPC marker), and CD29 (integrin *β*1/fibronectin receptor; Figure 2). NG2 expression was demonstrated immunocytochemically (Figure 3). Stem cell antigen-1 (Sca-1), CD34 (an hematopoietic and EPC marker), CD133 (a stem cell marker), CD31 (an endothelial marker), CD90 (THY1 T-cell antigen; a MSC marker), and CD105 (endoglin; a MSC marker) were expressed by small cell subsets. CD38, CD40, Flk-1, and major histocompatibility complex (MHC) class II molecules were not expressed. In cells cultured in MesenCult for 2 months and then changed to DMEM-10% FCS for 3 weeks, CD45^+^, CD11b^+^, or c-kit^+^ subsets were reduced by approximately half compared with cells maintained in MesenCult (Figure 4). Cell culturing in DMEM-10% FCS supplemented with GM-CSF did not alter CD45, CD11b, and c-kit expression (data not shown).

### 3.3. Nonclonal Sphere Formation

**Figure 5:**
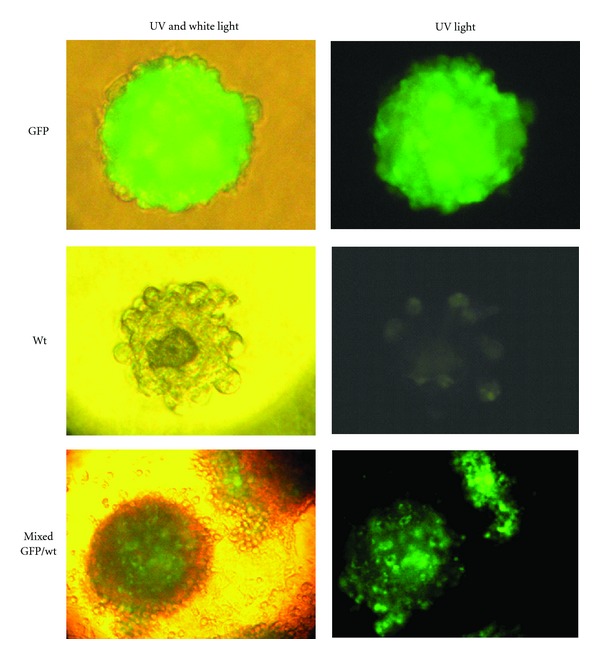
Photomicrographs and UV photomicrographs of sphere clusters formed by pancreas outgrowth-derived cells from either WT or GFP-transgenic mice, or by mixtures of the two. The latter show a patchy white/green pattern under the UV light, indicating that spheres are nonclonal.

The isolated population formed spherical clusters that detached from plastic and floated free in the medium. Spheres collected and plated onto new plates disaggregated and gave rise to a monolayer of sphere-derived cells, which were able to form a second generation of spheres. This procedure could be repeated for 3 cycles, at least. We tested the clonality of first-generation spheres by culturing mixtures of cells derived from GFP transgenic and WT mice. While spheres formed by GFP cells were entirely green fluorescent, and those formed by WT cells were non-fluorescent, those formed by mixtures of GFP and WT cells included both green fluorescent and non-fluorescent areas (Figure 5), indicating nonclonality.

### 3.4. 3D-Cell Culture System in MethoCult

**Figure 6:**
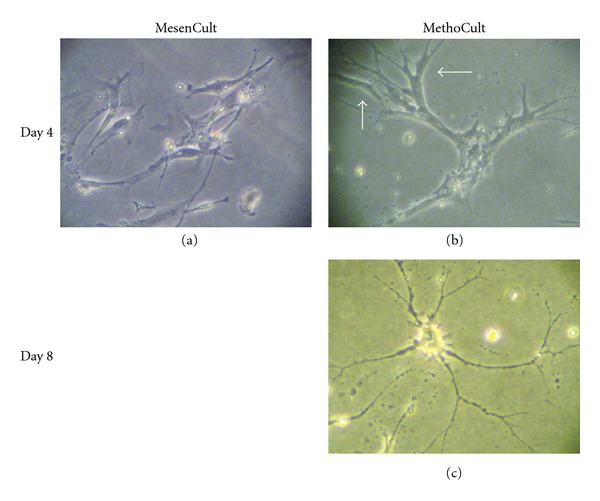
Pancreas-derived cells (5 weeks after plating; passage 3) were seeded at very low density (10^3^ cells/35 mm plate) in either MesenCult (a) or MethoCult (b and c). Pictures were taken 4–8 days later. Thin dendritic-like cell extensions, or filaments, were seen in MethoCult (c), but not in MesenCult (not shown).

Cells placed in a 3D-culture MethoCult system formed long, dendritic-like filaments after 4 to 8 days in culture (Figure 6).

### 3.5. Cell Differentiation Potential

**Figure 7:**
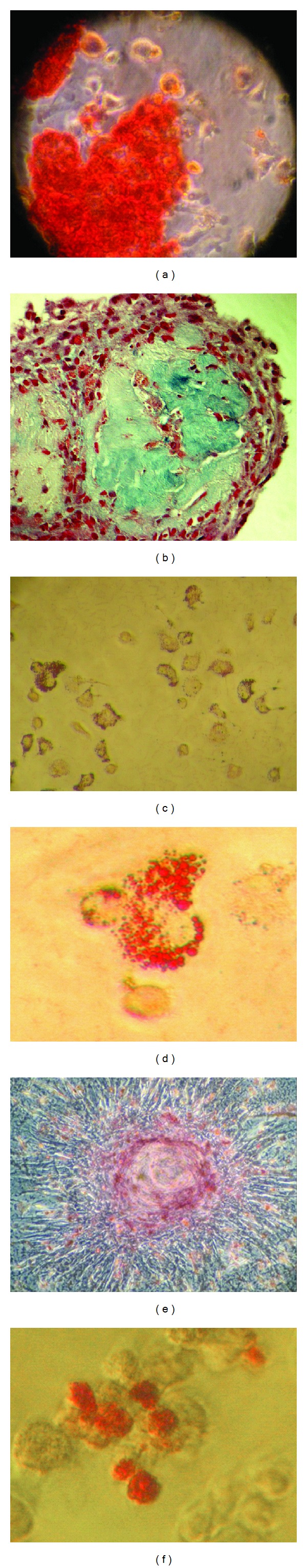
Multilineage differentiation of pancreas-derived cells. (a) Osteogenic differentiation (Alizarin-red staining). (b) Chondrogenic differentiation (Alcian-blue staining). (c and d) Adipogenic differentiation (Oil red-O staining; low/high magnification views). (e and f) Cells cultured for 2 months in MesenCult showed areas of positive staining with dithizone (red) as evidence of zinc-rich insulin-producing cells (low/high magnification views).

Under appropriate conditions, pancreas outgrowth-derived cells differentiated along osteogenic, chondrogenic, and adipogenic lineages, as evidenced by Alizarin-red (Figure 7(a)), Alcian-blue (Figure 7(b)), and oil red-O staining (Figures 7(c) and 7(d)), respectively. Dithizone staining was positive for a subset of cells cultured in 1.27 *μ*M dexamethasone/MesenCult for 18 days (Figures 7(e) and 7(f)). 

## 4. Discussion

The main finding of the present study is that cells obtained by outgrowth from murine pancreas explants in MesenCult give rise to a population of myelomonocytoid cells endowed with mesenchymal differentiation potential. These cells also stain positive with dithizone, a zinc-chelating agent commonly used to detect insulin-producing cells [46–49]. The endocrine differentiation potential of these cells is being addressed in an ongoing study. In the present study, we focus on their phenotype and MSC-like characteristics.

Monocyte-derived cells include macrophages, fibrocytes, dendritic cells, osteoclasts, and adipocytes. Monocytes, unlike macrophages and fibrocytes, do not express CD105 [50–52]. Because the isolated population lacks CD105 expression, it appears to have a monocytoid phenotype. This population meets only part of the minimal criteria for defining MSC established by the International Society for Cellular Therapy [53]: plastic-adherence in standard culture conditions and capacity to differentiate into osteoblasts, adipocytes, and chondroblasts *in vitro* (in the present study, multilineage differentiation potential was not demonstrated with single-cell cloning). Regarding the cell-surface marker profile, the minimal criteria for defining MSC, namely, expression of CD105 and CD90 but not CD45, are not met. However, other MSC/stromal markers (CD44 and CD29) and pericyte/perivascular markers (NG2 and CD146) are expressed [42]. In this regard, it has been shown that human MSCs in several organs originate from pericytes/perivascular cells and express NG2 [54]. For comparison, previous studies have shown that human islet outgrowths are positive for multiple MSC and pericyte markers (CD105^+^, CD90^+^, CD44^+^, CD29^+^, NG2^+^, and CD146^+^) but negative for CD45.

 Pancreas-derived cells cultured in the 3D-MethoCult system exhibit a dendritic or oligodendrocytic-like morphology characterized by multiple branched filaments. Microglia have a CD11b^+^CD45^lo^ phenotype and can be distinguished from primary macrophages on the basis of their CD45 expression level [55]. Differentiation of mouse BM-derived stem cells toward microglia-like cells has been reported [56].

The isolated myelomonocytoid population appears to have an advantage in terms of survival or growth compared with other cells present in the cellular outgrowth from pancreatic explants. These cells may die off and be taken over by the myelomonocytoid component that persists after extended periods of time. The underlying mechanism is unclear. Because MesenCult is a commercially available medium that has been optimized for growth of MSC, the emergence of a myelomonocytoid population over time is somewhat surprising. In a previous study [15], we used this medium to expand mouse cardiac-derived MSC, which displayed a stable phenotype (Lin^−^, Sca-1^+^, CD90^+^, CD105^+^, CD45^−^, and CD31^−^) for more than 25 passages. This observation indicates that MesenCult can preserve the phenotype of cultured MSC for extended periods of time, at least under certain circumstances.

The mesenchymal differentiation potential of the myelomonocytoid population may appear at odds with the established CD45^−^ MSC phenotype [53]. However, Sordi et al. [41] recently showed that mesenchymal cells emerging from human pancreatic culture did not result from an epithelial to mesenchymal transition but represented the expansion of a pool of resident MSC located in the periacinar, perivascular, and periductal space. Using a GFP^+^ BM transplant model, they showed that mesenchymal cells emerging from pancreatic endocrine or exocrine tissue culture originated mainly from the CD45^+^ BM compartment. Athough these cells expressed negligible levels of islet-specific genes, they improved islet function and neovascularization after transplantation with a minimal islet mass in a mouse model. Kaiser et al. [57] showed that a small population of BM MSC originated from the CD45^+^CD34^+^ fraction, whereas the majority was obtained from the CD45^−^CD34^−^ fraction. MSC from either fraction could be differentiated into adipocytes, osteocytes, and chondroblasts. Additional studies confirmed that MSC can express CD45 under certain conditions [58–60]. In some cases, CD45 expression was dramatically downregulated during *in vitro* culture [57, 58]. As mentioned above, our data suggest that MesenCult may preserve CD45 expression in cultured pancreas-derived myelomonocytoid cells, whereas partial downregulation of CD45 expression was observed in a standard culture medium (DMEM-10% FCS).

Pancreas-derived myelomonocytoid cells form spherical clusters, which is considered a feature of stemness [43]. However, these spheres are nonclonal. Similar findings have been reported for neural stem cells, as colonies formed by these cells can grow clonal or nonclonal [61].

The origin of the isolated myelomonocytoid population remains unclear. It might originate from blood monocytes trapped in the intravascular compartment of tissue explants, as recently shown for CD45^+^ cells from cardiac explant outgrowths [62]. It should be mentioned, however, that we were not able to isolate myelomonocytoid cells from murine BM-derived cells using the same culture conditions. Alternatively, this population might originate from pancreas-resident monocytes, monocyte-derived cells, or MSC. In this regard, Freisinger et al. [63] showed that clonally isolated, adipose-derived MSC cultured in appropriate differentiation media gave rise to cells expressing monocyte/macrophage and early hematopoietic markers.

Our findings are in general agreement with recent reports on multipotent monocytes. Zhao et al. [64] isolated a subset of adult pluripotent stem cells (CD14^+^, CD34^+^, and CD45^+^) from human peripheral blood monocytes. These cells in appearance resembled fibroblasts, expanded in the presence of macrophage colony-stimulating factor (M-CSF), and could be differentiated into mature macrophages and T lymphocytes, as well as into epithelial, endothelial, neuronal, and liver cells in the presence of appropriate growth factors. Kuwana et al. [44] and Kuwana and Seta [65] described human blood monocyte-derived multipotent cells (MOMCs; CD14^+^, CD34^+^, CD45^+^, and type-I collagen^+^) that exhibited a fibroblast-like morphology and contained progenitors with the capacity to differentiate into bone, cartilage, fat, skeletal muscle, cardiac muscle, neuron, and endothelium [65–67]. Romagnani et al. [68] described circulating clonogenic, multipotent CD14^+^ CD34^lo^ cells that proliferated in response to stem cell growth factors. Ungefroren and Fändrich [69] reported that the programmable cell of monocytic origin (PCMO) is a potential adult stem/progenitor cell source for the generation of islet cells. Hur et al. [48] recently showed that human peripheral blood monocytes could be differentiated into insulin-producing cells using the hematosphere culture technique. Collectively, these data suggest that blood monocytes and monocyte-derived cells, although not considered classic adult stem cells, may represent versatile progenitor cells capable of generating multiple types of cells.

Owing to their mesenchymal differentiation potential, pancreas outgrowth-derived myelomonocytoid cells are of potential interest to cell therapy applications even though this aspect was not directly addressed by the present study. Sordi et al. [41] reported beneficial effects of pancreatic MSC in diabetic mice, as mentioned above. When cotransplanted with a minimal islet mass, these cells improved neovascularization and islet function. This effect was not due to MSC differentiation into insulin-secreting cells, but to MSC-mediated protective effects on transplanted islets. Moreover, Johansson et al. [70] showed that MSC within composite endothelial cell-MSC-pancreatic islets improved endothelial cell proliferation and sprouting *in vitro*. It therefore could be speculated that pancreas-derived myelomonocytoid cells endowed with MSC potential might exert trophic effects on pancreatic islets via paracrine mechanisms, as reported for pancreatic MSC by Sordi et al. [41]. Further studies are warranted to test this hypothesis and to define the origin and the endocrine potential of the pancreas-derived myelomonocytoid population.

## Supplementary Material

Table showing the primary antibodies used for flow cytometric analyses.
