# Supplementary material for: Characterization of Myelomonocytoid Progenitor Cells with Mesenchymal Differentiation Potential Obtained by Outgrowth from Pancreas Explants
Source: Biotechnol Res Int. 2012 Aug 21;2012:429868. doi: 10.1155/2012/429868 (PMC3431127; doi:10.1155/2012/429868)
Supplement: Supplementary file 1 — Table showing the primary antibodies used for flow cytometric analyses. [file 429868.f1.docx]

**Supplementary Table 1: Antibodies and reagents**

| **Specificity** | **Host/type** | **Clone (cat. Number)** | **Company** |
| --- | --- | --- | --- |
| anti-mouse CD11b–APC | rat monoclonal | M1/70.15.11.5 (130-091-241) | Miltenyi |
| anti-mouse CD29–PE | rat monoclonal | RTK2758 (400507) | BioLegend |
| anti-mouse CD31–APC | rat monoclonal | 390 (102409) | BioLegend |
| anti-mouse CD34–Alexa647 | rat monoclonal | RAM 34  (51-0341) | eBioscience |
| anti-mouse CD38–biotin | rat monoclonal | 90 (13-0381) | eBioscience |
| anti-mouse CD40–APC | rat monoclonal | 1C10 (17-0401) | eBioscience |
| anti-mouse/human CD44–APC | rat monoclonal | IM7 (103011) | BioLegend |
| anti-mouse CD45–APC | rat monoclonal | 30-F11  (17-0451) | eBioscience |
| anti-mouse CD45–FITC | rat monoclonal | 30-F11  (11-0451) | eBioscience |
| anti-mouse CD90.2–APC | rat monoclonal | 30-H12 (105311) | BioLegend |
| anti-mouse CD105–biotin | rat monoclonal | MJ7/18  (13-1051) | eBioscience |
| anti-mouse CD105–PE | rat monoclonal | (120407) | BioLegend |
| anti-mouse CD106–biotin | rat monoclonal | 429 (13-1061) | eBioscience |
| anti-mouse CD117/c-kit–APC | rat monoclonal | 3C1  (130-091-729) | Miltenyi |
| anti-mouse CD133–APC | rat monoclonal | MB9-3G8  (130-092-335) | Miltenyi |
| anti-mouse MHC Class II–APC | rat monoclonal | M5/114.15.2 (17-5321) | eBioscience |
| anti-mouse Sca-1–APC | rat monoclonal | D7 (17-5981) | eBioscience |
| anti-mouse differentiation lineage–biotin | rat | (130-092-613) | Miltenyi |
| anti-NG2 chondroitin sulfate proteoglycan | rabbit polyclonal | (AB5320) | Chemicon/Millipore |
| anti-mouse Flk-1/CD309 | rat monclonal | Avas12a1  (13-5821) | eBioscience |
| allophycocyanin-conjugated streptavidin |  | (17-4317) | eBioscience |
| anti-biotin–FITC | mouse monoclonal | Bio3-18E7  (130-090-857) | Miltenyi |
| rat IgG2a isotype control–biotin | rat monoclonal | eBR2a(13-4321) | eBioscience |
| rat IgG2a isotype control–PE | rat monoclonal | eBR2a(12-4321) | eBioscience |
| rat IgG2a isotype control–FITC | rat monoclonal | eBR2a(11-4321) | eBioscience |
| rat IgG2a isotype control–APC | rat monoclonal | eBR2a(17-4321) | eBioscience |
| rat IgG2a,κ isotype control | rat monoclonal | R35-95 (559073) | BD |
| rat IgG2a,κ isotype control–PE | rat monoclonal | 400507 | Biolegend |
| mouse IgG1,κ isotype control–APC | mouse monoclonal | P3 (17-4714) | eBioscience |
| mouse IgG1,κ isotype control | mouse monoclonal | MOPC-31C  (557273) | BD |
